# Family and Personal Adjustment of Economically Disadvantaged Chinese Adolescents in Hong Kong

**DOI:** 10.1100/2012/142689

**Published:** 2012-08-01

**Authors:** Daniel T. L. Shek, Pik Fong Tsui

**Affiliations:** ^1^Department of Applied Social Sciences, The Hong Kong Polytechnic University, Hong Kong; ^2^Public Policy Research Institute, The Hong Kong Polytechnic University, Hong Kong; ^3^Department of Social Work, East China Normal University, Shanghai 200241, China; ^4^Kiang Wu Nursing College of Macau, Macau; ^5^Division of Adolescent Medicine, Department of Pediatrics, Kentucky Children's Hospital, University of Kentucky College of Medicine, Lexington, KY 40506-9983, USA

## Abstract

This study attempted to examine the relationship between poverty and adolescent developmental outcomes in the family and personal domains in 3,328 Chinese secondary school students in Hong Kong. Developmental outcomes included positive youth development constructs, problem behaviors, perceived family interaction, and parental parenting. Results showed that adolescents experiencing poverty did not differ from nonpoor adolescents in terms of risk behavior and in most indicators of positive youth development. On the other hand, adolescents with economic disadvantage displayed lower levels of positive identity, family interaction, and perceived paternal parenting than did those without economic disadvantage.

## 1. Introduction

In 2009, Hong Kong was ranked the first in wealth disparity in the world [1]. The poorest 10% of people shared 2% of the territory's wealth while the richest 10% of the people possessed 34% of Hong Kong's wealth. The wealth gap between the poor and the rich is becoming severe. In the fourth quarter of 2009, the household median income in Hong Kong was HK$17,500 (roughly equivalent to US$2,244). Hong Kong has no “official” poverty line, but there is a so-called “safety net” for the poor. The comprehensive social security assistance (CSSA) is a welfare scheme of the Hong Kong Government for people whose income is insufficient to satisfy their basic needs. According to the figures reported by the Hong Kong Census and Statistics Department, there were a total of 130,900 children and adolescents aged 6–14 living in households with income below CSSA in 2009 [2].

What is the effect of poverty? In the multidimensional perspective proposed by the United Nations [3], what matter most of poverty is “a focus on the opportunities—such as a set of endowments and access to markets—that are available to people. If an individual does not possess sufficient endowments or capabilities, such as a basic education, or does not have the opportunity to acquire them, he or she will have a limited ability to escape poverty” (page 9). Opportunities are especially important to adolescents who are undergoing intensive development in different physical and psychosocial domains. A review of the literature reveals that a variety of mechanisms linked socioeconomic status to child development. In particular, poverty adversely affects children's cognitive, social, psychological, academic, behavioral, and emotional development [4–6]. For example, Leung and Shek [7, 8] argued that economic disadvantage impaired family processes which in turn would negatively impact adolescent development.

 Various studies have shown that adolescent mental health problems were associated with poverty. Eamon [9] studied a sample of 898 young adolescents and found that poverty predicted young adolescent depressive symptoms. Similar results were found in the longitudinal study conducted by Najman et al. [10]. The research examined 2,609 adolescents aged 14–21 who provided self-report data on their level of anxiety and depression. Results indicated that family poverty led to higher rates of adolescent anxiety and depression. After examining 1,704 low-income adolescents, Hammack et al. [11] reported that poor adolescents were more likely to exhibit symptoms of depression, anxiety, hostility, and aggression.

 Identity formation is a very important stage of adolescents. After reviewing the literature on identity and poverty, Phillips and Pittman [12] argued that poverty had a negative impact on identity processes of adolescents and pointed out that “stress, social stigma or marginalization, and the nature of the opportunity structures faced by many poor adolescents conspire to create a context that is not conducive to exploring identity issues” (page 123). The study conducted by Crocker and Major [13] indicated that stigmatized groups generally had lower global self-esteem than did the nonstigma groups. Other studies also showed that poverty induced stigma on people [14]. Based on such research findings, it can be conjectured that social stigma associated with poverty makes adolescents feeling inferior to their economically advantaged counterparts.

 Limited opportunities brought forth by poverty also impair the future orientation of poor adolescents. McLoyd et al. [5] reviewed studies concerning the future orientations of adolescents and concluded that socioeconomic status and parenting style were important factors predicting future orientation. Poor adolescents were more aware of the limited life chances, and they were reported to have lower occupational aspirations and expectations as well as future orientations compared to economically advantaged adolescents.

 Apart from poorer mental health, adolescents in poverty were also found to have higher propensity to delinquent behaviors [15, 16], such as substance abuse, sex-related problems, school failure, and school dropout. Using structural equation models, Brook et al. [17] found that family poverty was associated with poor parent-child relationship which finally contributed to the risky sexual behavior in South African adolescents. Moreover, poor neighborhoods and feelings of hopelessness associated with economic disadvantage also increased the tendency of delinquent behaviors among poor adolescents [18]. Research suggests that poverty has a significant direct effect on adolescent antisocial behavior and that parent-child conflict, neighborhood problems, and deviant peer pressure are significant mediators [19]. 

 According to the family stress model, it is proposed that “economic hardship adversely affects children's psychological adjustment indirectly through its impact on the parent's behavior toward the child” [5, page 451]. Research studies showed that dimensions of family functioning were correlated with adolescent psychological well-being [20, 21]. Hammack et al.'s [11] study found that family stress was a mediator between poverty and depressed mood in low-income African-American adolescents. In the Chinese context, several studies examined the relationship between poverty and adolescent development outcomes. Shek [22] studied 3,017 Hong Kong secondary school students with and without economic disadvantage and found that perceived paternal behavioral control and father-child relational qualities were more negative in poor students than in nonpoor group students. Besides, students experiencing economic disadvantage also had poorer psychological well-being than their wealthier counterparts. In another study, Shek [23] investigated perceived family functioning and family adjustment in Chinese adolescents with economic disadvantage. Results showed that perceived family functioning was related to adolescent psychological well-being (existential well-being, life satisfaction, self-esteem, and general psychiatric morbidity) and problem behavior (substance abuse and delinquency). However, contrary to existing literature, Kwan [24] found that adolescents experiencing economic disadvantage in Hong Kong had better mental health than did economic advantaged respondents. Besides, he did not find any relationship between economic well-being and life satisfaction.

 Although poverty is a hot issue in the territory in the past decade which can be reflected from the establishment of the Commission on Poverty and the Community Care Fund, Hong Kong lacks comprehensive study on the influence of poverty on adolescent development. Focusing on adolescents' potentials instead of their deficits is a current trend, and researchers have put efforts in evaluating the effects of positive youth development programs toward adolescent development [25]. As indicated by McLoyd et al. [5], “despite strong scholarly interest in understanding positive youth development and finding ways to promote it, empirical work on these issues specifically in relation to youth who are poor or from low SES (socioeconomic status) backgrounds is very sparse.” [5, page 477]. Against this background, this study tried to examine if adolescents with and without economic disadvantage differ in their different adjustment domains, including personal domain (such as different positive youth development constructs including bonding, resilience, social competence, emotional competence, cognitive competence, behavioral competence, moral competence, self-determination self-efficacy, beliefs in the future, clear and positive identity, prosocial norms, prosocial involvements, and spirituality) and family process (including family functioning and parental control). The findings reported in this paper were derived from the first wave of a six-year longitudinal study that was designed to investigate different developmental domains of adolescents in Hong Kong. Because a large volume of data has been generated from this study, the primary focus of this paper is placed on the difference between adolescents with and without economic disadvantage on personal adjustment (positive youth development constructs and problematic behavior) and family processes (family functioning and parental control).

## 2. Methods

The present study is part of a large longitudinal study aiming at tracking the developmental trends based on different positive youth development indicators and risk behaviors among Hong Kong adolescents over time. A total of 28 secondary schools in Hong Kong were randomly selected to participate in the study. In this paper, data pertinent to the relationship between economic situation of the respondents and adolescent development in the first wave of a six-year longitudinal are presented.

### 2.1. Participants and Procedures

All Secondary 1 students in the selected school were invited to complete a questionnaire anonymously. A total of 3,328 students recruited from 28 secondary schools responded to the questionnaire (mean age = 12.59 years, SD = 0.74). These included 1,719 boys, 1,572 girls, and 37 students did not indicate their gender. Most students were born in Hong Kong (78.1%), 19.9% of the participants came from Mainland China, and 2.0% were from other places. The background demographic information of the participants is summarized in Table 1. Students were asked about their family financial conditions. As stated before, as Hong Kong has no poverty line, respondents whose families were receiving comprehensive social security assistance (CSSA) were categorized as the poor group (*N* = 225) while those who did not receive CSSA formed the nonpoor group (*N* = 2,606). For the remaining 465 respondents, they did not indicate whether they were receiving CSSA and thus their data were not included in this study.

 Data collection was conducted by a trained research assistant in classroom settings with standardized instructions. At each measurement occasion, the purposes of the study were introduced and confidentiality of the data collected was repeatedly ensured for all participants. School, parent, and student consent had been obtained prior to data collection. Participants responded to the questionnaires in a self-administered format with sufficient time given. The questionnaire took roughly 30 to 45 minutes to complete. The research assistant was present throughout the administration process to answer possible questions from the participants.

### 2.2. Instruments

In the school year of 2009-2010, the participants responded to a comprehensive youth development questionnaire including both existing instruments and scales developed by the first author. Participants were invited to respond to a composite questionnaire asking them about different aspects of their development. The following only highlights those measures related to the present study.

#### 2.2.1. Participants' Demographic Information

Questions on gender, age, place of birth, number of family members, parents' marital status, parents' educational level, and family financial situation were asked.

#### 2.2.2. Chinese Positive Youth Development Scale (CPYDS)

The CPYDS is an instrument assessing different positive youth development constructs. It consists of 15 subscales which include bonding (BO), resilience (RE), social competence (SC), recognition for positive behavior (PB), emotional competence (EC), cognitive competence (CC), behavioral competence (BC), moral competence (MC), self-determination (SD), self-efficacy (SE), clear and positive identity (SI), beliefs in the future (BF), prosocial involvement (PI), prosocial norms (PN), and spirituality (SP). Each construct has three items in the questionnaire. Except spirituality which is a measure of a 7-point scale, all other constructs assess the respondents in a 6-point Likert scale (1 = strongly disagree to 6 = strongly agree). The higher scores in the scale denote higher levels of psychosocial competence. Details of the items can be seen in Shek et al. [26]. Using multigroup confirmatory factor analyses (MCFA), Shek and Ma [27] showed that the 15 basic dimensions of the CPYDS could be subsumed under four higher-order factors, including cognitive-behavioral competencies (CBC), prosocial attributes (PA), positive identity (PIT), and general positive youth development qualities (GPYDQ). Evidence of factorial invariance, in terms of configuration, first-order factor loadings, second-order factor loadings, intercepts of measured variable, and intercepts of first-order latent factor, was found. In short, existing research findings showed that the CPYDS is a valid and reliable instrument. These four composite indicators were used to assess the participants' positive youth development in the present study. The mean scores of the four indicators ranged from 1 to 6 with higher scores representing high competence in the constructs. Descriptive statistics about all variables under study are listed in Table 1.

#### 2.2.3. Delinquent Scale (DE)

The respondents were asked if they had performed the following problem behaviors and the frequency they performed such behaviors in the past one year on a six-point Likert scale (0 = never, 1 = one to two times; 2 = three to four times; 3 = five to six times; 4 = seven to eight times; 5 = nine to ten times; 6 = more than ten times): stealing, cheating, truancy, running away from home, damaging others' properties, assault, having sexual intercourse with others, gang fighting, speaking foul language, staying outside the home overnight without parental consent, strong arming others, and trespasses [28].

#### 2.2.4. Substance Use Scale (DRUG)

Eight items were used to assess the participants' frequency of using different types of substance in the past half a year, including alcohol, tobacco, ketamine, cannabis, cough mixture, organic solvent, pills (including ecstasy and methaqualone), and heroin. Participants rated the occurrence of these behaviors on a six-point Likert scale (0 = never; 1 = 1-2 times; 2 = 3–5 times; 3 = more than 5 times; 4 = several times a month; 5 = several times a week; 6 = everyday).

#### 2.2.5. Family Functioning

Family functioning domains including communication, conflict, and harmony of respondents were assessed by 9 items. This 9-item measure is a simplified version of the Chinese Family Assessment Instrument developed by the first author [29]. In the present study, three subscales, including mutuality (mutual support, love, and concern among family members), communication (frequency and nature of interaction among family members), conflicts and harmony (presence of conflicts and harmonious behavior in the family), were examined. A higher total score on the subscales indicated a higher level of positive family functioning.

#### 2.2.6. Paternal Parenting (PPALL)

There were 17 items assessing paternal parenting, including paternal knowledge (“My father clearly knows my situation in my school”; “My father clearly understands who my friends are”), paternal expectation (“My father requires me to have good behavior in school”; “My father has explicit requirements about how I make friends with others”), paternal monitoring (“my father actively understands my situation in school”; “my father takes initiatives to understand who my friends are”; “my father actively understands what I do after school”), satisfaction with paternal control (“I feel that how my father disciplines me is reasonable”; “I am glad to fulfill my father's expectations about me”; “I believe how my father disciplines me is beneficial to me”), paternal psychological control (“My father always wants to change my thoughts”; “My father thinks that his thoughts are more important than my thoughts”; “my father wants to control everything in my life”; “My father always wants to change me to fit his standard”), and father-child relationship (“I'm very satisfied my relationship with my father”; “I actively share the things that happen in my life with my father”; “I actively share my feelings with my father”). Participants rated the paternal parenting in a 4-point Likert scale (1 = totally disagree; 2 = disagree; 3 = agree; 4 = totally agree). Reliability analysis of the 17 items showed that this scale was reliable (alpha  =  .88).

#### 2.2.7. Maternal Parenting (MPALL)

Identical items for paternal parenting were used to assess the maternal parenting, including maternal knowledge (2 items), maternal expectation (2 items), maternal monitoring (3 items), satisfaction with maternal control (3 items), maternal psychological control (4 items), and mother-child relationship (3 items). Participants rated their relationship with their mothers in a 4-point Likert-scale (1 = totally disagree; 2 = disagree; 3 = agree; 4 = totally agree). Reliability analysis of the 17 items showed that this scale was reliable (alpha  =  .87).

## 3. Results

An examination of the characteristics of the poverty and nonpoverty groups showed that there was no difference between the two groups in terms of gender ratio. Yet, there were significant differences between the two groups in terms of age (*M *= 12.77, SD =  .84 for the poor group and *M *= 12.55, SD =  .70 for the nonpoor group; *t* = 4.47, *P* < .0001). Because of the possible confounding effect of age, multivariate analysis of covariance was performed to examine the differences between the poor group and nonpoor group on the developmental variables to control for the effect of age.

 Tables 2 and 3 present the occurrence of problem behavior and substance abuse behavior in the poor and nonpoor groups. For the differences between the two groups on different problem behaviors, a multivariate analysis of variance (MANCOVA) with poor and nonpoor groups as the main factor and age as the covariate was performed. No significant difference was found between the two groups in terms of problem behavior and substance abuse behavior.

 Regarding positive youth development qualities, a MANCOVA was carried out with the poor group versus the nonpoor group as the independent variable, the scores of CPYDS subscales (CBC, PA, PIT, GPYDQ) as dependent variables, and age as the covariate (see Table 4). There was a significant difference between poor and nonpoor groups on the combined dependent variables: *F*(4,2306) = 4.3, *P* < .01, Wilks' Lambda =  .99, partial eta squared =  .007. When the dependent variables were examined separately with the Bonferroni adjustment (*P* = .013), the only difference found was in the score of PIT (positive identity): *F*(1,2310) = 13.18, *P* < .001, partial eta squared =  .006. PIT is the mean score of beliefs in the future (BF) and clear and positive identity (SI). The mean scores indicated that the poor group reported a lower level of PIT (*M* = 4.03, SD = 1.09) than did the nonpoor group (*M* = 4.30, SD =  .93).

Regarding differences between the two groups on family processes (family interaction, paternal parenting, and maternal parenting), a MANCOVA was performed with poor group versus nonpoor group as the independent variable, the family interaction, paternal parenting, and maternal parenting as the dependent variables, and age as the covariate (see Table 4). Results showed that the effects for the three dependent variables together were significantly related to groups, *F*(3,2172) = 20.65, *P* < .001. When the results for the dependent variables were examined separately, significant group effects were found in both family interaction and paternal parenting. The mean scores indicated that the poor group had lower scores on family interaction (*M* = 3.56, SD =  .84) than did the nonpoor group (*M* = 3.78, SD =  .84). The poor group also had worse perceived paternal parenting (*M* = 2.28, SD =  .73) than did the nonpoor group (*M* = 2.63, SD =  .52). The effect size of the differences ranged from low to moderate levels.

## 4. Discussion

The present study attempted to find out the relationship between poverty and adolescent developmental outcomes, parental control, and family communication. Compared to adolescents experiencing economic disadvantage, adolescents not experiencing economic disadvantage had higher scores in PIT (i.e., positive identity). This finding is consistent with the Western literature which suggests that adolescents in poverty are more likely to be pessimistic about their future lives [30]. It can be argued that poverty leads adolescents fall into a spiraling circle regarding their hope for the future. As pointed out by Eamon [9], “lowered aspirations among poor youth may result from realistic appraisals of available opportunities and experiential recognition of the limited lives of the adults around them, but at the same time lowered aspirations may result in self-imposed limitations that further reduce opportunities. In addition to having low aspirations, poor youth also tend to have low occupational and educational expectations” (page 117).

In line with other studies [13, 14] that poverty was found to be directly linked to social stigma, the findings in the present study also showed a similar phenomenon that adolescents in hardship do have poorer identity. As existing studies mainly used negative indicators such as internalizing and externalizing behavior in understanding the impact of poverty on adolescent development, it is necessary to examine the influence of economic disadvantage on other aspects of positive youth development in future studies. In this study, no significant difference was found between the poor group and nonpoor group in terms of problem behaviors. The result is echoing the findings from other studies using the same subjects [31–33] stating that socioeconomic status is not a predictor for problematic behaviors, consumption of pornography materials, and internet addiction.

Having reviewed the conceptual and methodological issues in studying the relationship between adolescent development and economic disadvantage, Leung and Shek [7, 8] proposed a number of future research directions which include the identification of protective factors among poor adolescents and the incorporation of the cultural dimension to capture the ideological ingredients. Moreover, many researchers try to find out the mediating factors between poverty and adolescent developmental outcomes [10, 15, 18]. Thus, it would be interesting if we can test the mediating factors of adolescent developmental outcomes for this group of Chinese adolescents as well as to find out those protective factors of poor adolescents in Hong Kong in future studies. Besides, it is important to look at the moderating effect of poverty on adolescent developmental outcomes. Furthermore, some studies focus on the prolonged effect of poverty on children and adolescents [34]. As the present study is the first wave of a six-year longitudinal study, it would be more promising if data analyzed can be done for different waves to evaluate the prolonged effect of poverty on Chinese adolescents over time.

 Regarding the family factors, there were significant differences between the two groups in family interaction and paternal parenting, and these results were in line with Shek's studies [22, 23]. Fathers in the poor group were found to be perceived as poorer in parenting. It might be because fathers were viewed as the bread winners in traditional Chinese families and depending on CSSA for a living may cause stress between fathers and children. Moreover, as stated by Shek [22], poor fathers “might be blamed for causing poverty in the family, their children might perceive their parental control attributes and parent-child relational qualities negatively” (page 185).

 There are several strengths of this study. First, a large sample was involved which was randomly selected from schools in Hong Kong. Second, validated instruments were used to assess individual and family processes. Third, a wide range of personal and family adjustment measures were included in the study. Despite these strengths, several limitations of the study should be noted. First, caution must be made about the operational definition and classification of “poor” adolescents in the present study. As stated before, we simply categorized those respondents who had received CSSA as the poor group, but this may not be the best classification. According to the statistics of the Hong Kong Council of Social Services [35], 25.8% of children aged 6 to 14 could be regarded as living in poverty when using the household income as an indicator. Nevertheless, the poor group in the present study only accounted for 7.9% of the total respondents. As suggested by Wadsworth et al. [16], “there is growing consensus that SES (socioeconomic status) is best computed from parental education, occupational status, and family income” (page 160). We may get a clearer picture about the performance in different positive youth development constructs of adolescents in hardship if we can take into account other criteria for defining poverty. The authors have attempted to calculate the family household income in this study. However, due to too much missing data (because students may not be clear about their family financial situation), we could only use CSSA as a criterion for defining the poor group. Nevertheless, it is noteworthy that eligibility for comprehensive social security assistance is the “official” definition of poverty is Hong Kong. Second, only data reported by the students were collected in this study. It would be more comprehensive if we can get parents' views when analyzing parental control and family interaction. Third, as there are no conclusive findings on gender differences in the impact of poverty on adolescent development, gender was not included as a covariate in the present study. As such, this point should be taken into account in future studies.

Despite the above limitations, the present study gives us some idea about the performance of poor adolescents in different positive youth development constructs. There is a need to find out some means to alleviate the adverse effects of poverty on adolescents. School-based positive youth development programs such as the Project P.A.T.H.S. in Hong Kong may be a way out because there is evidence showing that universal positive youth development programs can help enhance different psychosocial competencies of participants [36–38] and at the same time not imposing any stigmatization effect on them. As stated before, research investigating the relationship between positive youth development and poverty is rare and the present study can be viewed as an addition to the existing literature, especially in the Chinese context.

## Figures and Tables

**Table 1:** Descriptive statistics about participants.

Categorical variables	*n*	%	
Gender	
Male	1,719	52.2%	
Female	1,572	47.8%	
Place of birth	
Hong Kong	2,590	78.3%	
Mainland China	655	19.8%	
Others	64	1.9%	
Family economic status	
Receiving CSSA	225	6.8%	
Not receiving CSSA	2606	78.3%	
Others	465	13.9%	
Receiving school textbook assistance scheme	
Full grant	368	11.61%	
Half grant	771	24.32%	
Not receiving any grant	2,031	64.07%	

Continuous variables	Mean	SD	Range

Age	12.59	0.74	10–18
CBC	4.45	0.75	1–6
PA	4.50	0.89	1–6
GPYDQ	4.50	0.71	1–6
PIT	4.24	0.96	1–6

Notes: CSSA: comprehensive social security assistance; CBC: cognitive behavioral competence; PA: prosocial attributes; GPYDQ: general positive youth development; PIT: positive identity.

**Table 2:** Past year exposure to substances.

	Never (%)		Attempted (%)	
	Poor group	Nonpoor group	Poor group	Nonpoor group
(1) Smoking	90.1	95.3	9.9	4.7
(2) Drinking	70.7	71.3	29.3	28.7
(3) Use ketamine	99.6	99.8	0.4	0.2
(4) Use cannabis	99.6	99.8	0.4	0.2
(5) Use cough medicine without coughing	99.6	99.3	0.4	0.7
(6) Use organic solvent	99.1	97.7	0.9	2.3
(7) Use pills (e.g., ecstasy)	100.0	85.0	0	15.0
(8) Use or inject heroin	100.0	96.1	0	3.9

**Table 3:** Delinquent behaviors between poor and nonpoor groups in the past year.

	Never (%)		Attempted (%) (1–4 times)		Attempted (%) (5 times or above)	
	Poor group	Nonpoor group	Poor group	Nonpoor group	Poor group	Nonpoor group
(1) Stealing	86.5	90.3	12.7	9.0	1.0	0.7
(2) Cheating	38.1	38.4	43.1	42.2	18.8	19.4
(3) Truancy	95.9	97.2	3.2	2.1	0.9	0.7
(4) Running away from home	94.1	96.7	5.4	3.0	0.5	0.3
(5) Damaging others' properties	88.8	86.1	10.3	12.1	0.9	1.8
(6) Assault	90.5	88.6	7.2	9.3	2.3	2.1
(7) Having sexual intercourse with others	98.2	99.6	1.8	0.3	0	0.1
(8) Group fighting	96.3	97.1	2.3	2.5	1.4	0.4
(9) Speaking foul language	24.9	30.3	37.1	38.0	38.0	31.7
(10) Staying outside overnight without parents' consent	97.2	97.2	2.3	2.1	0.5	0.7
(11) Strong arming others	79.1	85.0	16.8	11.3	4.1	3.7
(12) Trespasses	96.8	96.1	3.2	3.2	0	0.7

**Table 4:** Differences between poor group and nonpoor group in positive youth development constructs.

Measures	Poor group		Nonpoor group		*F* value
	Mean	SD	Mean	SD	
CBC	4.37	0.83	4.45	0.74	2.50
PA	4.45	0.93	4.53	0.87	1.70
GPYDQ	4.51	0.75	4.59	0.71	2.36
PIT	4.04	1.08	4.27	0.94	11.82*

Problem behavior	0.41	0.42	0.39	0.46	0.03
Substance abuse	0.08	0.19	0.09	0.21	0.44

Family interaction	3.48	0.83	3.78	0.81	25.28*
Paternal parenting	2.29	0.72	2.63	0.52	64.35*
Maternal parenting	2.86	0.58	2.93	0.50	4.32

Note. An overall alpha level based on the Bonferroni adjustment was carried out to adjust for inflated type 1 error.

*P < .01.
